# Comparison of the prediction accuracy of machine learning algorithms in crosslinguistic vowel classification

**DOI:** 10.1038/s41598-023-42818-3

**Published:** 2023-09-20

**Authors:** Georgios P. Georgiou

**Affiliations:** 1 Department of Languages and Literature, University of Nicosia, Nicosia, Cyprus; 2 Director of the University of Nicosia Phonetic Lab, Nicosia, Cyprus

**Keywords:** Human behaviour, Auditory system, Machine learning

## Abstract

Machine learning algorithms can be used for the prediction of nonnative sound classification based on crosslinguistic acoustic similarity. To date, very few linguistic studies have compared the classification accuracy of different algorithms. This study aims to assess how well machines align with human speech perception by assessing the ability of three machine learning algorithms, namely, linear discriminant analysis (LDA), decision tree (C5.0), and neural network (NNET), to predict the classification of second language (L2) sounds in terms of first language (L1) categories. The models were trained using the first three formants and duration of L1 vowels and fed with the same acoustic features of L2 vowels. To validate their accuracy, adult L2 speakers completed a perceptual classification task. The results indicated that NNET predicted with success the classification of all L2 vowels with the highest proportion in terms of L1 categories, while LDA and C5.0 missed only one vowel each. Furthermore, NNET exhibited superior accuracy in predicting the full range of above chance responses, followed closely by LDA. C5.0 did not meet the anticipated performance levels. The findings can hold significant implications for advancing both the theoretical and practical frameworks of speech acquisition.

## Introduction

Machine learning is regarded as a subset of artificial intelligence since it allows for the identification of significant patterns from examples provided during training. Over the last decade, the use of machine learning techniques has been extended to the prediction of listeners’ nonnative speech perception patterns. This is based on the either direct or indirect assumptions of several speech models (e.g., Speech Learning Model/SLMr^1,2^; Perceptual Assimilation Model/PAM-L2^3,4^; Second Language Linguistic Perception model^5^; Universal Perceptual Model^6^) that acoustic/articulatory-phonetic similarity between first language (L1) and second language (L2) sounds can predict the perception of L2 sounds. For example, Georgiou^7^ reported that the classification of English /ɪ/ and /iː/ in terms of Cypriot Greek /i/ and the poor discrimination of this contrast were successfully predicted by a machine learning classifier. Poor discrimination was also reflected in the speakers’ production patterns. Therefore, by training machine learning algorithms with L1 acoustic features and feeding them with the same features of a nonnative language, researchers can estimate perceptual and potentially production patterns of nonnative speech.

Linear Discriminant Analysis (LDA) is a classifier aiming to discover a linear conversion that enhances the distinguishability between different classes in the reduced dimensional space^8^. If the assumptions of normality and homoscedasticity are met, LDA serves as the most efficient Bayes classifier for binary classification^9^. LDA has been used as a tool for predicting the mapping of nonnative sounds in terms of the listeners’ L1 categories in a number of studies (e.g.,^10–14^). For instance, Gilichinskaya and Strange^14^ examined the perceptual assimilation of American English vowels to the L1 vowel categories of inexperienced Russian listeners. The predictions conducted using an LDA paradigm estimated successfully the assimilation of all but one vowel. In a more recent study, after training an LDA algorithm with the *F1*, *F2*, and duration of Cypriot Greek vowels, Georgiou^13^ found that the algorithm predicted accurately the classification of the majority of English vowels with the highest proportion in terms of L1 vowel categories. However, the power of the model significantly dropped in terms of predicting the full range of above chance responses.

C5.0 is another machine learning classification algorithm appearing as the extension of C4.5, which emerged from the Iterative Dichotomiser 3 algorithm^15^. The classifier is modelled on a structure that resembles a tree and seeks to find the best feature of a training test, leading to the division of the dataset into subsets. The process terminates at a specific branch and produces a leaf node that represents a classification, thereby allowing the researcher to evaluate the quality of the parameters and the contribution of the input features. Apart from decision trees, the algorithm includes rule-based models. C5.0 also uses a boosting technique to improve the performance of the model. Boosting involves repeatedly applying the C5.0 model to the training dataset, with an emphasis on misclassified records in subsequent iterations^16^. This iterative process can help decrease prediction errors related to bias and variance^17^. C5.0 was tested only in a few acoustic studies. For example, in a crossdialectal classification study, Themistocleous^18^ observed that the classification accuracy of C5.0 trained on the formant dynamics of vowel *F1*, *F2*, *F3*, *F4*, and duration outperformed the accuracy of LDA and Flexible Discriminant Analysis.

Artificial Neural Networks are machine learning algorithms mimicking the structure of the brain^19^. Each neuron in the network has the capability to receive and process an input signal and transmit an output signal^20^. Feedforward neural networks are among the most popular types of neural networks and are distinguished by the fact that information flow is unidirectional as there are no loops or cycles. The processing elements are organized into separate layers, where each of them receives input from the previous layer and transmits its output to the subsequent layer^21^. The first layer is the input layer that receives the raw input data, the last layer is the output layer that produces the predicted output, and the between layers are the hidden layers that are responsible for transforming the input data into a more meaningful representation. Neural network algorithms have not been extensively used in linguistics, at least inside the realm of theoretical linguistics. Neural networks have played a significant role in practical applications like speech recognition systems, natural language processing, and phoneme classification (e.g.,^22^), prioritizing real-world performance and efficiency. Balaji et al.^23^ concluded that an artificial neural network exhibited the best prediction accuracy for EEG-based unspoken speech and language classification among other classifiers. Some nonlinguistic studies support that NNET can achieve high prediction accuracy. For instance, Bataille et al.^24^ found that fluid responsiveness prediction in severe sepsis or septic shock could be predicted with high accuracy by both NNET and LDA.

This study aims to assess how well machines align with human speech perception. The following research question will be answered: Can machine learning algorithms, when trained on crosslinguistic acoustic data, achieve levels of accuracy in classifying L2 sounds that are comparable to the perceptual performance of L2 human listeners? For this purpose, a human classification test and three machine learning classifiers, namely, discriminant analysis, decision tree, and neural network were used. The three classifiers present with substantial differences in the way they conduct calculations and process the datasets. LDA is a widely used paradigm in the area of crosslinguistic sound classification due to its simplicity and capacity to produce accurate predictions. C5.0 is a decision tree algorithm, which can offer powerful predictions of sound classification, although its effectiveness has only been tested in a limited number of crosslinguistic studies. NNET is a relatively new and more sophisticated algorithm, which resembles the structure of the brain. This algorithm is not popular in crosslinguistic speech acquisition, but it is able to designate more complex relationships and provide more accurate predictions. To the author’s knowledge, this is the first study investigating the accuracy of different machine learning algorithms in predicting the classification of L2 sounds in terms of L1 categories as previous work in this area has predominantly employed the LDA paradigm. The selection of the algorithm that provides optimal predictions is of paramount importance as it can inform current speech acquisition theories, enhancing our understanding of the role of acoustic cues in L2 speech perception. In addition, it can facilitate the modelling of listeners’ L2 sound discrimination difficulties, offering important pedagogical implications as well as improvement of speech technology. The study’s protocol was based on the collection of speech samples targeting the L1 and L2 vowels and the extraction of their acoustic characteristics. Then, machine learning classifiers were trained on the L1 acoustic characteristics and the testing data was later added to the models. Finally, an L2 vowel classification task was completed by human participants to assess the predictions of the algorithms.

## Methodology

All experimental protocols were approved by the Ethics Committee of the Department of Languages and Literature, University of Nicosia. All methods were carried out in accordance with ethical standards as laid down in the 1964 Declaration of Helsinki and its later amendments or comparable ethical standards. Participation was voluntary and participants could leave the experiment at any stage. The collected data remained under the possession of the researcher and were not disclosed. Each participant was identified using codes to ensure anonymity. Informed consent was obtained from all subjects.

### Extraction of speech features

The training data included the *F1*, *F2*, *F3*, and duration of Cypriot Greek vowels /i e a o u/ produced by 22 (*n*_females_ = 11) adult Cypriot Greek speakers. The vowels were embedded in a /pVs/ (where V is the target vowel) context and were part of the carrier phrase ‘Léne < target word > tóra’ (‘They say < word > now’). The testing data included the same acoustic parameters for Standard Southern British English vowels /ɪ iː e ɜː æ ɑː ʌ ɒ ɔː uː ʊ/ produced by 20 (*n*_females_ = 10) adult English speakers. They were included in an /hVd/ context and were part of the carrier phrase “They say < word > now”. All participants were asked to utter the phrases with a normal speaking rate and their productions were recorded using a professional audio recorder at a 44.1 kHz sampling rate. The target vowels were embedded in different word context in the two languages. Acoustic measurements of vowels, such as formant frequencies and duration, can be influenced by phonetic context. While this is an important consideration for certain research questions, this study aimed to investigate the classifiers’ ability to generalize across phonetic contexts, which may be more relevant for real-world applications. In addition, in terms of the interpretation of results, the goal was not to claim absolute universality in vowel classification but rather to evaluate the models’ performance under varying phonetic contexts, which provides valuable information for applications like speech recognition systems. Finally, matching all aspects of stimulus materials, including consonantal context, across the two languages was challenging since real words were included to collect precise acoustic features of the L1 sounds.

The speakers’ output was sent to Praat^25^ for speech analysis. The following adjustments were made: windows length: 0.025 ms, pre-emphasis: 50 Hz, and spectrogram view range: 5500 Hz. To extract formant frequencies, the initial point of vowels’ acoustic analysis was considered as the end of the quasi-periodicity of the preceding consonant /p/ for Cypriot Greek and /h/ for English and the onset point of vowel (V), while the ending point of vowels’ acoustic analysis was considered as the end of the quasi-periodicity of vowel (V) and the onset point of the second consonant /s/ for Cypriot Greek and /d/ for English. Formants were measured at their midpoint and normalized through the *vowels* package^26^. The Lobanov method was used, which relies on the *z*-score formula *Z* = *(F ─ mean)/SD*, where F is the formant of a particular vowel, mean is the value for that particular formant for a given speaker, and SD is the standard deviation for the speaker’s particular formant^27^. Vocalic duration was extracted using manual labelling of the starting and ending points of each vowel token and derived from the measurement of the interval between the starting and ending points of the vocalic part.

### Machine learning algorithms

**Figure 1:**
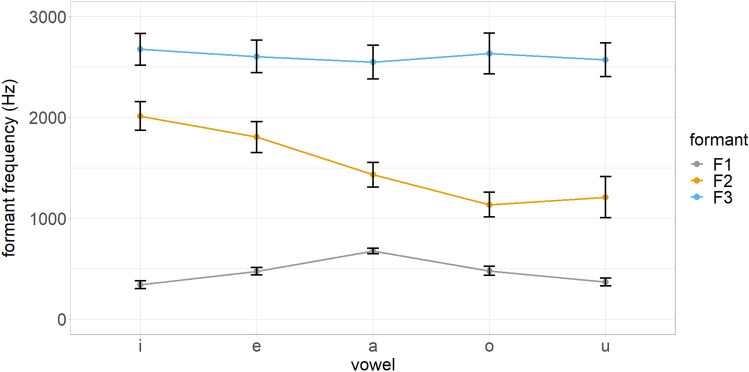
Formant frequencies (scaled) of vowels in the trained model.

**Figure 2:**
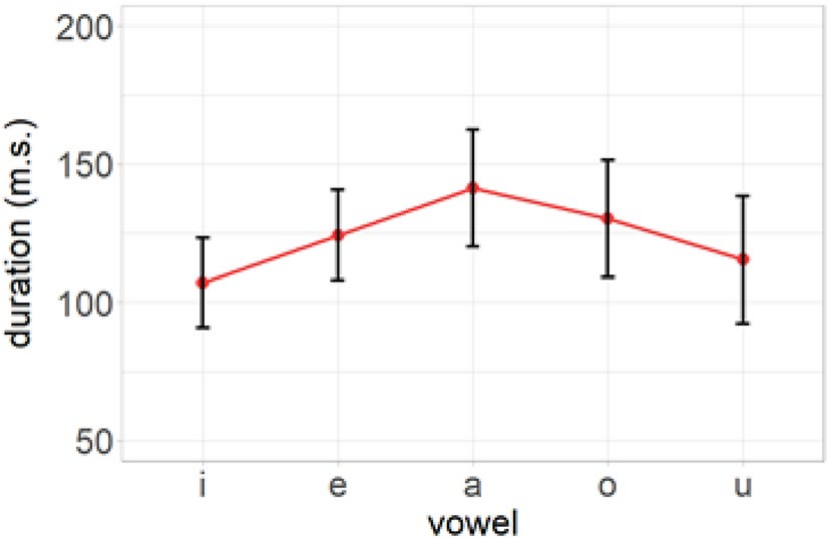
Duration of vowels in the trained model.

Three separate machine learning classification algorithms, namely LDA, C5.0, and NNET were used to predict the classification of L2 sounds in terms of L1 phonetic categories on the basis of crosslinguistic acoustic similarity. The classifiers were trained in R^28^ using the MASS^29^, C5.0^30^, and nnet^31^ packages respectively. The training set included the normalized *F1*, *F2*, *F3*, and duration of Cypriot Greek vowels, while the testing set included the same values for English vowels, which were used to feed the trained models. There were 44 training and 20 test samples per vowel class. The training corpus consisted of 50% of the data, while the testing corpus consisted of the remaining 50%. The crossvalidation method indicated a 0.94% prediction accuracy for the trained LDA model, which shows the proportion of correctly classified instances in the training dataset. The C5.0 model was trained using 10 folds and 3 repetitions and adjusted to automatically select the optimal boosting parameter value. The final trained model demonstrated 0.95% prediction accuracy. The NNET model was trained using the following parameters: size = 10, maxit = 1000, and decay = 0.9. The trained model presented a 0.95% prediction accuracy. The models’ optimization adjustments were crossvalidated using the exand.grid function. Figures 1 and 2 show the formant frequencies (in Hz) and duration (in ms) of vowels in the trained model.

### Perception study

#### Participants

The sample consisted of 20 Cypriot Greek speakers (*n*_females_ = 10) with an age range of 19–43 (*M*_*age*_ = 31.9, *SD* = 6.93). They were born, raised, and permanently resided in Cyprus at the time of the study and had a moderate socioeconomic status. The participants had never lived in an English-speaking country for a long time. They had an average English learning onset age of 8.35 (*SD* = 1.35), daily English use of 1.2 h (*SD* = 1.21), and daily English input of 3.05 h (*SD* = 1.99). All participants reported knowledge of English at the B2/C1 levels and rated their understanding skills as 4.55/5 (*SD* = 0.59). All of them reported healthy vision and hearing and the absence of any language or cognitive disorders.

#### Speech materials

The test stimuli included the 11 English monophthongs embedded in monosyllabic /hVd/ words as part of the carrier phrase “They say < word > now”. Two adult female English speakers were recorded in a quiet room producing the carrier phrases (44.1 kHz sampling rate). The speakers were instructed to produce the phrases as if speaking to another person. The output was digitized and sent to Praat to adjust for peak intensity. The vowels from the two female speakers of English were a subset of those fed to the three classifiers. Although nonidentical datasets were given to classifiers vs humans, this would not prevent the meaningful comparisons. This is because the speakers' L2 categories might be characterized by perceptual flexibility due to their experience with varied stimuli (e.g., different accents and pronunciations, different contexts, etc.), which allows them to employ more general categorization patterns. From a practical perspective, it is useful for the machine models to be trained on different acoustic features compared to the perceptual tests, taking into account that algorithms used in speech recognition systems are trained on different speech patterns than those encountered by individuals during speech perception. In addition, it is commonplace for several similar studies to employ a subset of the machine learning classifier data to feed the perceptual tests as it is not always feasible to use a large number of acoustic features.

#### Procedure

All participants completed the test individually in quiet rooms. The classification test was prepared in a Praat script, which was presented to the participants using a PC monitor. Participants were instructed to sit in front of the monitor and follow the instructions. They listened to the words that included the target English vowels through the headphones, and they were asked to click on the script label that was acoustically the most similar exemplar to the vowel they heard. The labels included the orthographic representation of the five Cypriot Greek vowels, namely, “ι”, “ε”, “α”, “ο”, and “ου”. Despite the lack of time restriction, participants were advised to provide quick responses to the script. In addition, there was an optional five-minute break at the midpoint. The speakers classified a total number of 44 trials each (11 vowels × 4 repetitions). The interval between a click and the presentation of the next trial was 500 ms. No feedback was given and there was no option for repetition of the acoustic stimuli. Prior to the main experiment, participants completed a familiarization test with four test items to ensure that they understood the experiment requirements.

#### Ethical approval

This study received ethical approval from the Ethics Committee of the Department of Languages and Literature, University of Nicosia. All participants gave their written consent for participation, according to the Declaration of Helsinki.

## Results

### Machine learning algorithm predictions

**Figure 3:**
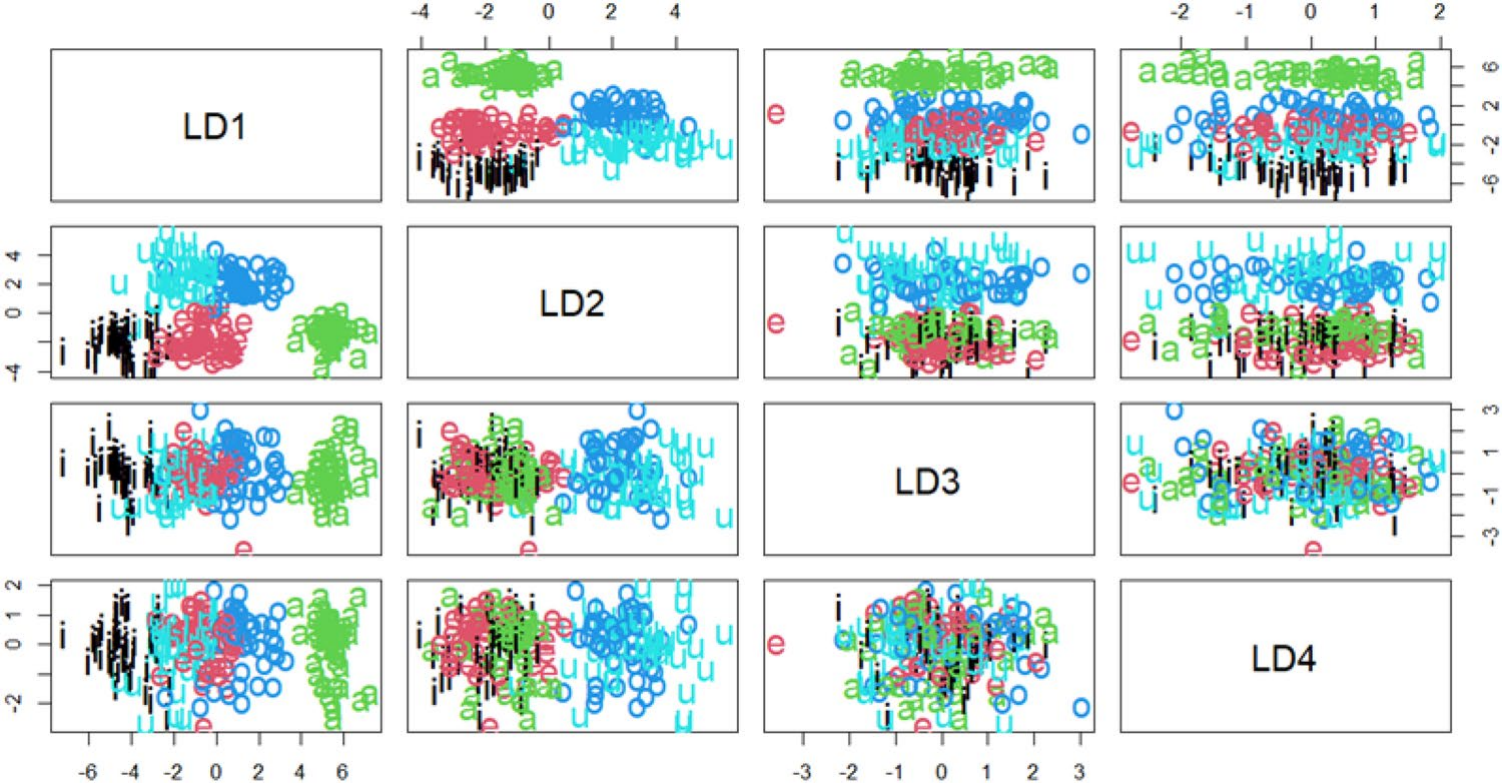
Linear Discriminant Analysis scatter plot for vowel classification. LDA1, LDA2, LDA3, and LDA refer to the different linear discriminants (i.e., *F1*, *F2*, *F3*, and duration) that are used to discriminate between the vowel classes.

**Figure 4:**
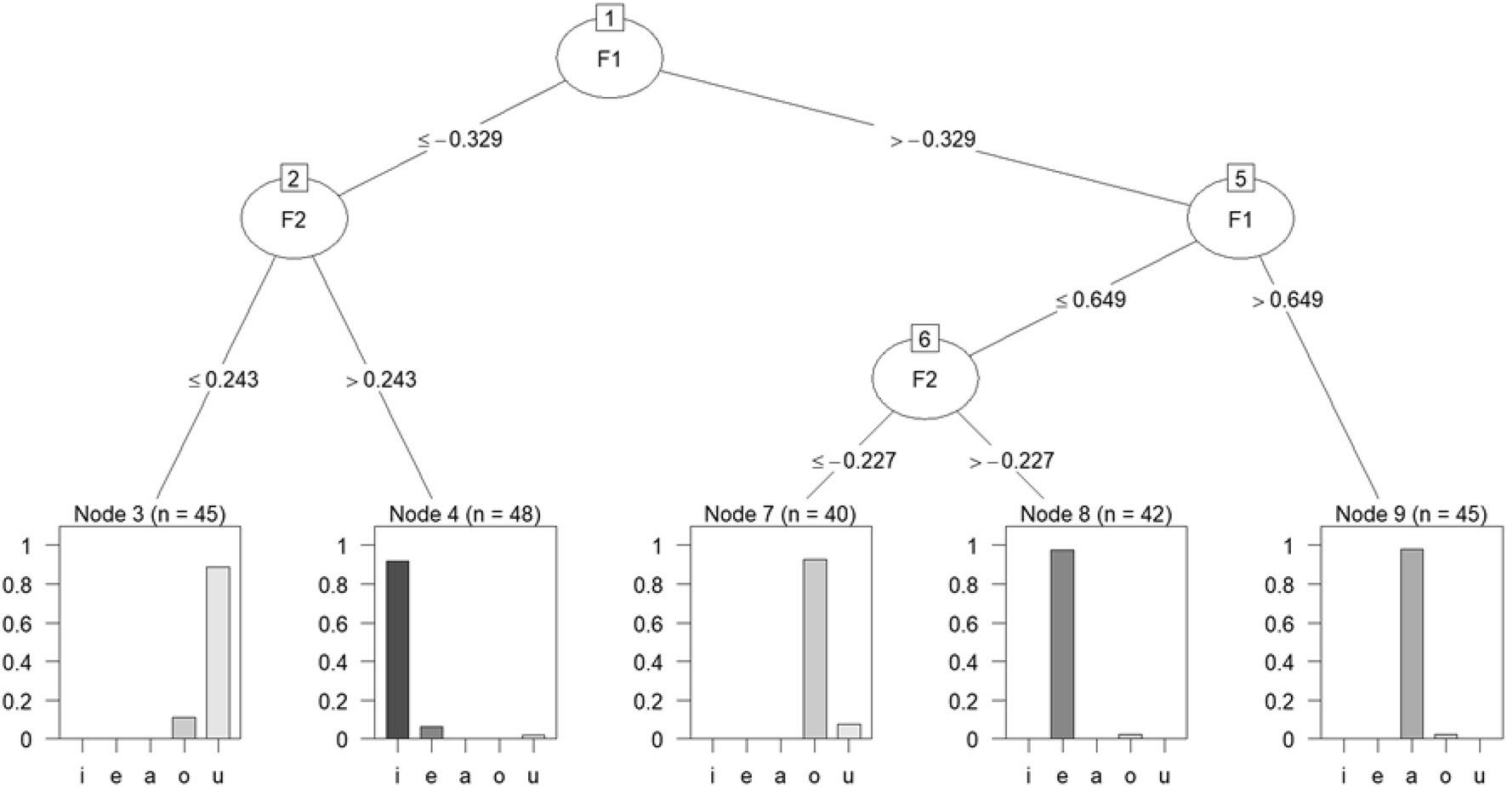
Decision tree for the C5.0 algorithm. Each node represents a decision based on a particular acoustic feature (e.g., *F1*, *F2*, etc.). The branches indicate the possible paths the data can take based on the feature values. Leaf nodes represent the final outcome or prediction of the model.

**Figure 5:**
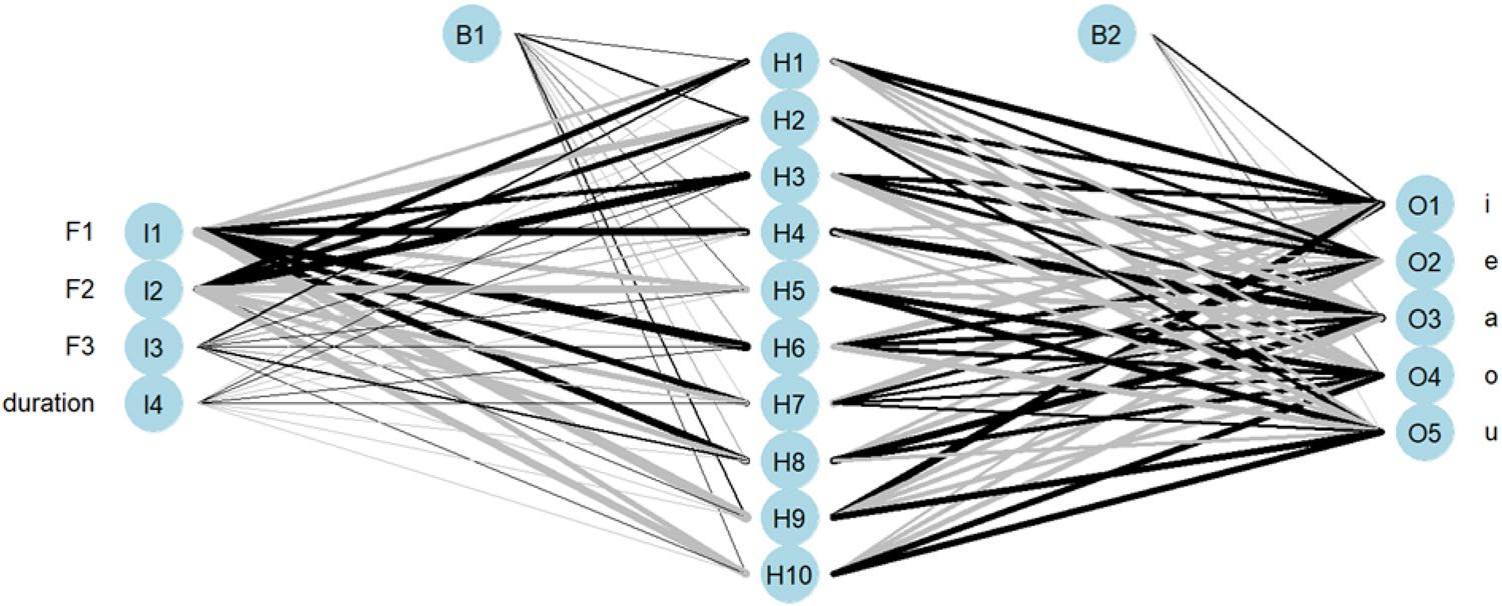
NNET architecture plot for the trained vowels. I, B, H, and O represent the input layers, bias units, hidden layers, and output layers respectively. Each input is connected to every hidden neuron, which is represented by the lines, and the hidden neurons are further connected to the outputs.

The three algorithms classified the L2 English vowels in terms of L1 vowel categories. All classifiers exhibited similar behaviour for English /ɪ/ and /iː/ as both vowels were classified as L1 /i/. Although English /e/ was classified with the highest proportion as L1 /e/ in all algorithms, some classifications occurred in terms of L1 /a/ in C5.0. English /ɜː/ and /æ/ were consistently classified as L1 /e/ and /a/ in all classifiers. English /ɑː/ was highly classified as L1 /a/ in all algorithms; however, it was classified to some extent as L1 /o/ in LDA and NNET. English /ʌ/ had high classification in terms of L1 /a/ in all classifiers, while there were some classifications in terms of L1 /o/ in LDA. English /ɒ/ was classified as an instance of L1 /o/ in all classifiers, while English /ɔː/ was mainly classified as L1 /u/ in LDA and C5.0 but as L1 /o/ in NNET. English /ʊ/ was consistently classified as L1 /u/ in all algorithms. English /uː/ despite being classified as L1 /u/ in all algorithms, some classifications also occurred in terms of L1 /i/ in LDA and C5.0. Overall, in terms of responses with the highest proportion, LDA and C5.0 exhibited 100% agreement, LDA and NNET 90.9% agreement, and C5.0 and NNET 90.9% agreement. In terms of the full range of above chance responses (those ≥ 0.20, given that there are five L1 Cypriot Greek vowels) together with the responses with the highest proportion for each English vowel, LDA and C5.0 exhibited 63.6% agreement, LDA and NNET 72.7% agreement, and C5.0 and NNET 63.6% agreement. The detailed confusion matrix of the classification accuracy across all classifiers is shown in Table 1. The plots of the LDA, C5.0, and NNET algorithms are illustrated in Figs. 3, 4, and 5 respectively.

**Table 1:**
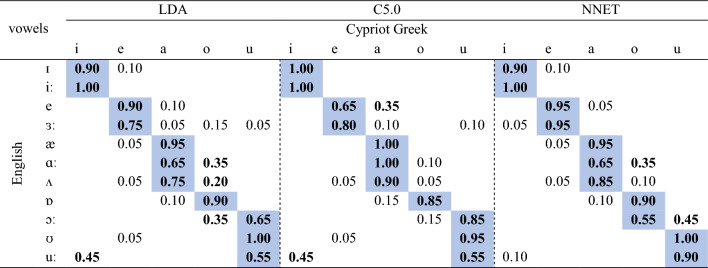
Classification of English vowels in terms of Cypriot Greek categories based on LDA, C5.0, and NNET algorithms. Blue cells represent vowels classified with the highest proportion. Bold indicates above chance classifications (≥ 0.20).

### Perception study

The perceptual test revealed the classification patterns of the L2 speakers. English /ɪ/ and iː/ were classified as L1 /i/, while /e/ and /ɜː/ were classified as L1 /e/. English /æ/ was perceived as an acoustic exemplar of L1 /a/. English /ɑː/ and /ʌ/ were mostly classified as L1 /a/ and to a lesser extent as L1 /o/. English /ɒ/ and /ɔː/ were highly classified as L1 /o/, while they were also above chance instances of L1 /a/ and /u/ respectively. English /ʊ/ and /uː/ were both classified as L1 /u/. Table 2 presents the classification of English vowels in terms of L1 categories as elicited from the perceptual test.

**Table 2:**
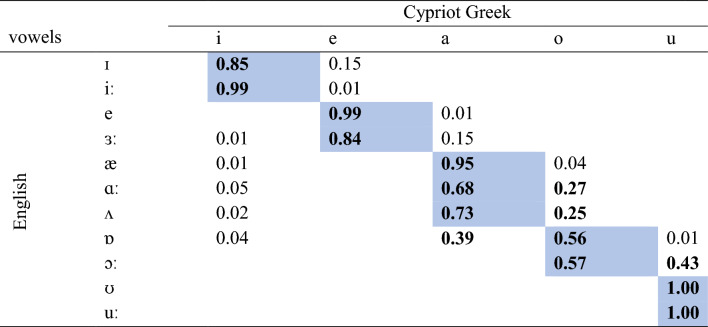
Human classification of English vowels in terms of Cypriot Greek categories. Blue cells represent vowels classified with the highest proportion. Bold indicates above chance classifications (≥ 0.20).

**Figure 6:**
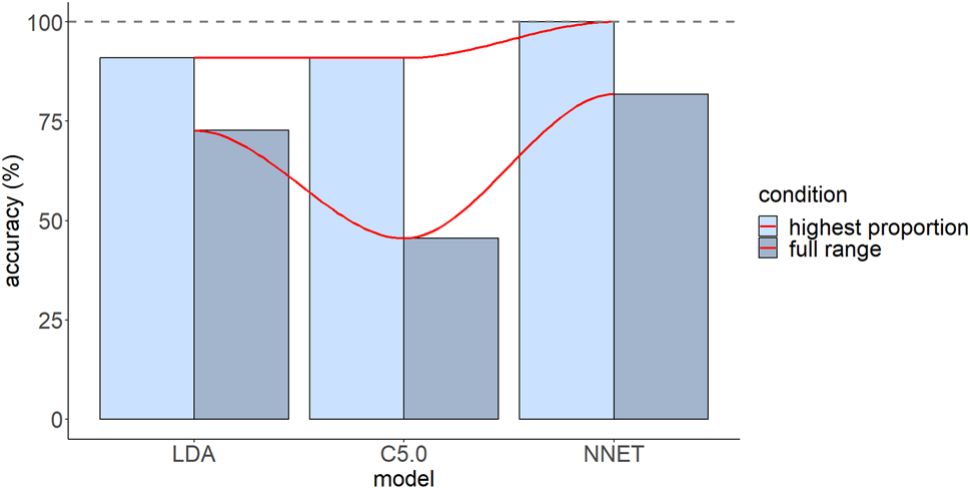
The classification accuracy predictions of LDA, C5.0, and NNET.

The classifications of the three machine learning algorithms can be compared to those of human participants. With respect to the responses with the highest proportions in terms of a single L1 response, LDA and C5.0 exhibited 90.9% prediction accuracy, managing to predict the classification of all English vowels but /ɔː/. In contrast, NNET exhibited 100% prediction accuracy, as it predicted the classification of all English vowels. With respect to the prediction of the range of above chances responses of a certain English vowel in terms of a single L1 vowel together with the responses with the highest proportion, LDA presented with 72.7% prediction accuracy as it did not estimate accurately the classification of /ɒ/, /ɔː/, and /ʊ/. C5.0 presented with 45.5% prediction accuracy as it did not predict accurately vowels /e/, /ɑː/, /ʌ/, /ɒ/, /ɔː/, and /uː/. NNET presented with 81.8% prediction accuracy, failing to predict the classification of English vowels /ʌ/ and /ɒ/. Overall, NNET is the most accurate model both in terms of predicting classifications with the highest proportion and predicting the full range of above chance responses for a particular L2 vowel. LDA still maintains good discrimination accuracy for the aforementioned parameters, while C5.0 can only accurately predict classifications with the highest proportion. The accuracy of the models is shown in Fig. 6.

## Discussion

This study aimed to examine whether machine learning algorithms such as LDA, C5.0, and NNET trained on crosslinguistic acoustic data can achieve levels of accuracy in classifying L2 sounds that are comparable to the perceptual performance of L2 human listeners. The algorithms were trained in R with the first three formant frequencies and duration of Cypriot Greek vowels and the testing data included the same acoustic features of English vowels. A group of Cypriot Greek speakers of English completed a perceptual classification test, the results of which were used as a baseline for the evaluation of the machine learning algorithm predictions.

The results demonstrated good overall performance for LDA and NNET and poor for C5.0. The good accuracy of LDA is also supported by previous evidence. For example, several crosslinguistic studies (e.g.,^10,13^) found that this algorithm could predict quite successfully the mapping of L2 sounds to L1 categories. However, the prediction accuracy is reduced in terms of predicting the full range of above chance responses^13,32^. Thus, when demands grow with the calculation of the range of responses, the power of the classifiers is diminished. This is consistent with the results of this study as the prediction accuracy of all models dropped in this condition. The smallest decrease was reported for NNET followed by LDA and C5.0. In addition, in alignment with the results of this study, the literature suggests that NNET demonstrates better performance in group prediction compared to other traditional approaches^33,34^. Other studies reported that both NNET and LDA offer comparable predictions. For example, Bataille et al.^24^ found that both algorithms predicted fluid responsiveness as the standard approach. Similarly, Finch and Schneider^35^ observed that NNET behaves similarly to LDA for group classification. These results partially corroborate the results of this study as the better performance of NNET compared to LDA was confined to the classification of one L2 vowel only. Furthermore, C5.0 did not accurately estimate the full range of above chance responses. Along the same lines, Bataille et al.^24^ and Finch and Schneider^35^ argued that decision tree algorithms had a lower rate of correct classification compared to other approaches such as NNET and LDA.

The better performance of NNET can be explained on the basis of its benefits compared to the other algorithms. NNET employs deep learning techniques with the use of training models including multiple neuron layers. Such an architecture can capture abstract and high-level features, granting it an advantage over simpler algorithms such as LDA and C5.0. NNET’s abilities are not restricted just to the examination of simple interactions but to the inspection of various combinations of predictors by creating hidden nodes as weighted outputs of many variables^35^. In addition, NNET is characterized by high flexibility and adaptability since its internal parameters can be adjusted to better align with the underlying patterns inherent in the dataset^36^. Furthermore, taking into account that linear boundaries between categories in n-dimensional spaces do not always apply in human speech perception and that NNET has the ability to grasp complex and nonlinear relationships (e.g., see^19,37^), its better performance over LDA, which is based on linear relationships, is somehow expected. However, the performance of LDA was just below that of NNET. There might be several interpretations for this. For instance, the complexity of the relationship between L1 and L2 categories might not be highly nonlinear, thereby explaining the relatively similar accuracy of the two algorithms. It should be taken into account that several factors that can increase nonlinearity (e.g., L2 learning onset age, L2 input, L2 use, etc.) were controlled in this study to obtain a sample with similar characteristics. In addition, the small size of the datasets may have played a role in the models’ performances. NNET provides optimized results when large datasets are used, while the use of small datasets may inhibit its ability to learn complex patterns^38^; therefore, the algorithm’s great advantage over LDA is diminished. Finally, C5.0 did not exhibit accurate estimations. This result may have emerged since this algorithm is prone to overfitting^39^, especially when the tree becomes too complex or the dataset is small. It could be also due to its incapability to deal with continuous variables just like those used in the study^40,41^.

Speech perception is a multifaceted process that takes place in the motor cortex^42^ and requires speakers to assign discrete boundaries to the perceived acoustic cues, leading to the development of different phonetic categories for various sounds (for categorical perception, see^43^). Speakers rely on their L1 categories to perceive nonnative speech sounds and therefore the nonnative sound acoustic characteristics are filtered through the properties of the acoustically most similar sounds of their L1. Artificial intelligence mimics the ability of humans to classify nonnative speech sounds by leveraging various machine learning algorithms and training datasets, which take into consideration both the L1 and L2 sound acoustic characteristics. To match human classification as precisely as possible, the artificial models need to be trained with a greater number of acoustic features, which are available to humans. For example, Georgiou^13^ found that an LDA model trained on the *F1*, *F2*, and duration of Cypriot Greek vowels predicted the classification of only seven out of 11 English vowels with the highest proportion in terms of the speakers’ L1 categories. In contrast, Georgiou^12^ observed that an LDA model with *F1*, *F2*, *F3*, and duration as input measures predicted accurately the classification of 10 out of 12 Dutch vowels in terms of Cypriot Greek phonetic categories; this shows better performance compared to the previous study.

Cognitive models such as the Speech Learning Model, Perceptual Assimilation Model, Second Language Linguistic Perception model, and Universal Perceptual Model support that L2 speech patterns can be estimated by comparing the acoustic similarity between L1 and L2 sounds. Although the development of machine-based speech recognition or phonetic classification systems has been inspired by cognitive models, these two domains remain distinct in their goals and methodologies. Cognitive models contribute to our understanding of the mechanisms involved in language acquisition, while machine-based approaches primarily aim to develop practical tools for tasks like automatic speech recognition, phonetic analysis, and language processing. However, the models can inform the design of machine-based systems. For example, if specific sound contrasts are identified by a cognitive model to be challenging for L2 learners, adaptive algorithms that prioritize the training of these contrasts can be employed, resulting in an efficient and targeted learning intervention. Or, machines can be trained according to acoustic features extracted by a cognitive model that captures how similar an L2 speech sound is to an L1 sound. In addition, the theoretical predictions of the models can be developed based on machine learning classifications. Specifically, the discrimination of nonnative sound contrasts can be predicted on the basis of the classification conducted by a machine learning classifier. For example, Georgiou and Dimitriou^44^ found that the discrimination accuracy of four Dutch vowel contrasts could successfully be predicted using the classification results of an LDA algorithm and the predictions of the Universal Perceptual Model.

The findings can offer important insights into the formulation of predictions in speech perception studies. More specifically, more sophisticated algorithms can be employed such as neural networks for the estimation of L2 sound classification and the development of the research hypotheses. This is because they comprise more powerful tools compared to the traditional LDA methods, which have been extensively used in the past. Furthermore, while cognitive models and machine-based approaches, as previously mentioned, have distinct goals and methodologies, the latter can also offer valuable insights into a better understanding of the processes underlying speech perception (e.g., the role of acoustic cues such as formants and duration). Thus, these two approaches can be seen as complementary. In addition, the findings can inform pedagogy. By selecting a classifier that demonstrates optimal predictive performance, educators will be able to map the difficulties of learners with different L1 backgrounds as regards the perception of L2 sounds and therefore develop the appropriate educational tools and platforms to facilitate L2 learning. Educators may offer adaptive and personalized instruction based on the needs of individual learners who can receive tailored feedback, have access to customized materials, and track their progress in the learning of L2 pronunciation. Overall, the practical outcomes of this study may lead to the advancement of research in the area of L2 acquisition and the improvement of L2 teaching and learning.

In addition, there might be benefits to the improvement of automatic speech recognition systems (ASR). ASRs, which are used for the transcription of spoken language into text, usually need to recognize and understand speech from nonnative speakers. They should be able to identify subtle phonetic nuances and better adapt to variations in speech sounds as a result of the unique phonetic characteristics of nonnative speakers. The systems’ accuracy can be enhanced if they have improved capabilities of sound classification. There might also be potential benefits for other applications. For instance, voice assistants need to understand the individuals’ speech to respond to their queries. The same applies to chatbots that are widely used nowadays by companies and which need to respond to customers’ requests.

## Conclusions

The capacity of artificial intelligence modelling in predicting the classification of L2 sounds in terms of the listeners’ L1 categories was accurate in the NNET and LDA algorithms. This can have significant implications for crosslinguistic speech acquisition studies, which base their predictions on machine learning classifiers. Apart from the traditional LDA paradigms, neural network paradigms such as NNET can be used for crosslinguistic sound classification, which might be even more powerful than LDA. The findings from this study can also make a valuable contribution toward enhancing language learning and speech technology systems. Future studies may employ larger samples and compare the accuracy of different sets of classifiers.

## Data Availability

Data is available at https://osf.io/rvb8m/.
